# The most common aeroallergens in a tropical region in Southwestern Iran

**DOI:** 10.1186/1939-4551-6-7

**Published:** 2013-04-04

**Authors:** Mohammad-Ali Assarehzadegan, AbdolHossein Shakurnia, Akram Amini

**Affiliations:** 1 Immunology department, Faculty of Medicine, Jundishapur University of Medical Sciences, Ahvaz, Iran

**Keywords:** Aeroallergen, Prevalence, Allergy, Tropical, Sensitization

## Abstract

**Background:**

Respiratory allergies are the most important public health issues in the world. They are caused by aeroallergens which play great role in pathogenesis of respiratory allergic diseases.

**Methods:**

The current study was conducted to evaluate the prevalence of positive skin test for various aeroallergens among allergic patients in Ahvaz, southwest Iran. 299 participants with allergic rhinitis (seasonal or perennial) were selected. Skin prick test using twenty three common allergen extracts was performed on all patients.

**Results:**

The overall frequency of sensitization to any allergen was 85.6%. In outdoor allergens the most prevalent aeroallergen category was weeds (89%) followed by tree and grasses, and in indoor allergens, mites (43%) were the most prevalent aeroallergen. The mean and median numbers of positive test reactions among those with positive test responses were 11.5 and 13.0, respectively. 84% of patients were poly-sensitised and about 50% of them were sensitised to more than twelve different allergens.

**Conclusion:**

The results of the study revealed that prevalence of the skin prick reactivity to weed pollens is significant in southwest Iran and multiple sensitizations were common.

## Background

Respiratory allergies, particularly allergic rhinitis, are among the most common allergies within various populations all over the world and the incidence is expected to increase [1]. Prevalence of respiratory allergy has been determined between 10-30% in different studies [2-5].

Aeroallergens play great role in pathogenesis of respiratory allergic diseases. Pollens, molds and pets are the main sources of allergens [5,6]. The occurrence of allergic rhinitis varies among different countries, as well as different regions within a country, which could be related to the type of allergens existing in those regions [6-8]. Moreover, for an efficient diagnosis of the specific allergy and an efficient treatment, it is very important to identify common aeroallergens in the area [3,6,9].

Ahvaz, the capital of Khuzestan province, is located in the southwest of Iran with an approximate population of 1.4 millions (census 2006). Ahvaz has a desert climate with long, extremely hot summers and mild, short winters. It is consistently one of the hottest cities in Iran, with summer temperatures regularly at least 45 degrees Celsius, sometimes exceeding 50 degrees Celsius with many sandstorms and dust storms common during the summer period while in winters the minimum temperature could fall around +5 degrees Celsius. Winters in Ahvaz have no snow. The average annual rainfall is around 230 mm. In our region the dust storm was a usual phenomenon in some days in spring and summer but now it may occur almost all of the year [10-12].

Due to the lack of published studies that have been conducted on sensitization to aeroallergens in the southwest of Iran, the prevalence of common aeroallergens is still not fully understood in the region. The current study was therefore conducted to determine the prevalence of positive skin test for various aeroallergens among allergic patients in the city of Ahvaz. It was hoped that the results would be useful to health system policy-makers in planning respiratory allergic diseases prevention programs in the region.

## Methods

### Study population

This study was conducted as the cross-sectional study on candidates participating in Immunology department of Jundishapur University of Medical Sciences and Khuzestan Jahad Daneshgahi Medical Center from July 2010 to September 2011. Two hundred and ninety nine participants with allergic rhinitis (seasonal or perennial) were selected based on ISAAC [13] (The International Study of Asthma and Allergies in Childhood) questioner. The study was conducted according to good clinical practices and the Declaration of Helsinki. The Ethics Committee of Ahvaz Jundishapur University of Medical Sciences was approved the study protocol and an official agreement from all of patients was obtained before the study.

The diagnosis of current allergic rhinitis was established by clinical examination and a combination of having a problem with sneezing or a runny or blocked nose in the absence of a cold or flu and a positive SPT reaction (diameter >3 mm more than negative control) to at least one of the twenty three aeroallergens. A study questionnaire requesting demographic data, family history of atopy and respiratory symptoms was also administered to each patient.

### Skin prick test and total IgE

Skin prick test using twenty three common allergen extracts (HollisterStier, Spokane, WA, USA) was performed on all patients in accordance with previous study [6] (Table 1). In this study, allergens were selected based on the plant species existing in our region and other possible allergens were identified from consulting ear, nose and throat specialists [14]. Seven different types of weeds (*Amaranthus palmeri*, *Amaranthus retroflexus*, *Kochia scoparia*, *Chenopodium album*, *Salsola kali*, *Plantago lanceolata*, *Artemisia vulgaris*) and four grasses (*Poa pratensis*, *Cynodon dactylon*, *Lolium perenne*, *Sorghum halepense*) pollen extracts along with four trees (*Acacia longifolia*, *Prosopis juliflora*, *Eucalyptus globulus*, *Fraxinus americana*) extracts; four molds (*Alternaria* Mix, *Aspergillus fumigatus*, *Cephalosporium acremonium*, *Penicillium* Mix), two mites (*Dermatophagoides pteronyssinus*, *Dermatophagoides farinae*), house dust mix, and a mixture of two different cockroach (*Periplaneta americana and Blattela germanica*) extracts were used.

**Table 1:** Prevalence of positive skin prick test and total IgE among allergic rhinitis patients

**Aeroallergens**		**All patients (%)**	**Allergic rhinitis**		***p *value**
**Common name**	**Scientific name**		**Seasonal (%)**	**Perennial (%)**	
**Weeds**					
Russian thistle	*Salsola kali*	72.9	79.4	20.6	0.1
Pigweed	*Amaranthus retroflexus*	70.9	79.2	20.8	0.2
Carless weed	*Amaranthus palmeri*	68.6	79.5	20.5	0.1
Lamb’s quarter	*Chenopodium album*	67.9	79.3	20.7	0.2
Burning Bush	*Kochia scoparia*	66.6	79.9	20.1	0.1
Mugwort	*Artemisia douglasiana*	60.9	80.8	19.2	0.07
Plantain	*Plantago lanceolata*	55.2	80.0	20.0	0.2
**Any weeds**		89.0	79.4	20.6	0.09
**Grasses**					
Kentucky blue grass	*Poa pratensis*	54.8	81.1	18.9	0.08
Bermuda grass	*Cynodon dactylon*	52.5	80.9	19.1	0.1
Johnson grass	*Sorghum halepense*	46.5	80.6	19.4	0.2
Perennial rye grass	*Lolium perenne*	36.1	78.7	21.3	0.7
**Any grasses**		75.0	80.6	19.7	0.06
**Trees**					
Mesquite	*Prosopis juliflora*	65.9	80.7	19.3	**0**.**04**
White Ash	*Fraxinus americana*	52.5	80.9	19.1	0.1
Acacia	*Acacia longifolia*	48.2	81.9	18.1	0.07
Eucalyptus	*Eucalyptus globulus*	21.7	89.2	10.8	**0**.**009**
**Any Trees**		86.0	79.0	21.0	0.2
**Mites**					
Mite	*Dermatophagoides farinae*	32.1	76.0	24.0	0.6
Mite	*Dermatophagoides pteronyssinus*	27.1	69.1	30.9	**0**.**03**
**Any mites**		43.0	75.2	24.8	0.4
**Molds**					
Fungus	*Cephalosporium acremonium*	11.4	85.3	14.7	0.3
Fungus	*Aspergillus fumigatus*	5.0	80.0	20.0	0.5
Penicillium Mix^*^	Penicillium Mix	9.4	75.0	25.0	0.8
Alternaria Mix^†^	Alternaria Mix	8.0	75.0	25.0	0.7
**Any molds**		24.0	82.0	18.0	0.4
**House dust**					
House dust mix^‡^	House dust mix	19.1	82.5	17.5	0.3
**Cockroach**					
Cockroach mix	*P*. *americana* + *B*. *germanica*	30.8	75.0	25.0	0.4
**Total IgE** (mean, IU/ml)		177.77	172.54	196.39	0.3

^*^ The extract includes *P*. *digitatum*, *expansum*, *glaucum*, *roseum* and *notatum*. ^†^ The extract includes *A*. *tenuis* and *Hormodendrum cladosporioides*. ^‡^ Pooled collection containing kapok, feather mix (chicken, duck and goose) and mattress dust.

Skin prick tests were performed under physician’s supervision. In this test, allergen extracts were put on patients’ inner forearms and irritation of the epidermis was caused by prick method using the lancet and the result was observed after 15 minutes. Next, the diameter of patient’s skin reaction was measured and compared with negative (glycerin saline) and positive (histamine hydrochloride, 10 mg/ml) controls. Patients with a wheal diameter >3 mm were considered positive compared with negative and positive controls. Patients using drugs affecting skin test were excluded from the study.

In the end, enzyme immunoassay method (ELISA) was performed to collect the total IgE serum. Serum samples were stored at −20°C until the analysis. ELISA test was performed in duplicate by using the commercial kit (DIPLUS, Canada). Based on manufacturer’s instruction, samples with more than 100 IU/ml total IgE was considered elevated.

### Statistical analysis

In the end, all the data was analyzed by SPSS Version 18.0 software (Version 11.0 of SPSS software) (Chicago, USA). Chi-square test was used to compare the relationship between variables. *p* value less than 0.05 was considered significant.

## Results

Demographic characteristics of subjects are presented in Table 2. The mean duration of allergic rhinitis was 6.78 (±3.89) years. Symptoms of active conjunctivitis and allergic rhinitis were found in 172 (72.8%) of patients (Table 2). Among patients with positive for SPT, seasonal pattern was seen in 77.7% of the patients, perennial pattern in 22.3%. In addition, 193 patients (64.5%) had positive family history of allergy.

**Table 2:** Characteristics of patient population

**Characteristics**	**No**. **of cases (%)**
All patients	299 (100)
The mean duration of allergic rhinitis (±SD): 6.78 (±3.89)	
Gender	
Male	156 (52.2)
Female	143 (47.8)
Age (mean, range: 32.02, 4–70)	
Disease presentation	
Sneezing	237 (79.3)
Runny nose	254 (84.9)
Itching and nasal congestion	187 (62.5)
Rhinoconjuctivits	142 (47.5)
Allergic rhinitis (with skin prick test positive)	
Seasonal	199 (77.7)
Perennial	57 (22.3)
Family history of allergy	193 (64.5)

The overall frequency of sensitization to any allergen was 85.6% (256/299), whereas 14.4% (43/299) of patients did not react to any of the tested allergens.

In outdoor allergens the most prevalent aeroallergen category was weeds (89 %) followed by tree and grasses, and in indoor allergens, mites (43%) and cockroaches (30.1) were the most and least prevalent aeroallergen types, respectively (Table 1).

Prevalence of positive skin test to any allergen is shown in Table 1. Skin reaction to *Salsola kali* was the most common among the allergens (72.9%). Other prevalent weeds were *Amaranthus retroflexus* (70.9%), *Amaranthus palmeri* (68.6%), *Chenopodium album* (67.9%), and *Kochia scoparia* (66.6%). Ninety five percent of patients with positive skin prick test for outdoor allergens were also sensitized to at least to one of these five allergens, which belong to the Amaranthaceae and Chenopodiaceae families.

Among tree pollen, the most prevalent allergen was *Prosopis juliflora* with 65.9% and the least prevalent was *Eucalyptus globulus* with 21.7%.

Among indoor allergens the most prevalent allergen was *Dermatophagoides farinae* (32.1%). Skin reaction to the mixture of cockroach extracts of Americanus and Germanium has been found in 30.8% of patients.

**Figure 1:**
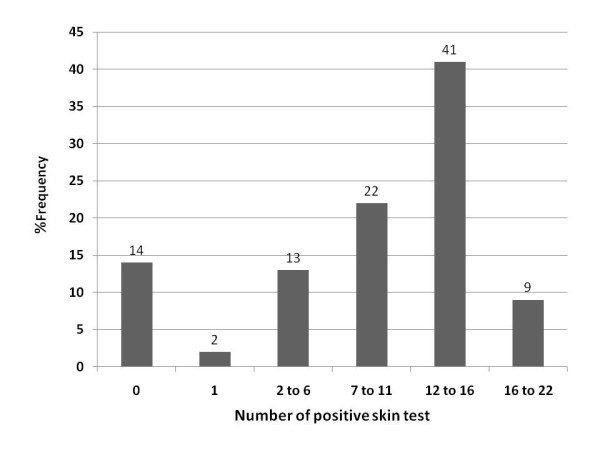
Percentage of patients according to number of positive skin test reactions.

The mean and median numbers of positive test reactions among those with positive test responses were 11.5 and 13.0, respectively. The mean age of men and women with positive SPT were 33.69 ± 14.61 and 31.92 ± 13.05, respectively. However, the differences were not significant (*p*=0.9). Eighty four percent of patients were poly-sensitised (positive skin reaction to more than two allergens) and about 50% of them were sensitised to more than twelve different allergens (Figure 1).

As shown in Table 3, the analysis of the positive skin prick test rate according to age groups revealed, with the exception of the grasses pollen allergens, there is no significant difference between sensitization to selected allergens among 4 age groups and for 4–17 age group compared with 31–45 age groups.

**Table 3:** Age distribution of patients with positive skin prick test to indoor and outdoor allergens

**Allergens**	**% Frequency of positive skin prick test**				
	**4-17 yr (n= 40 )**	**18-30 yr (n= 76)**	**31-45 yr (n=96)**	**>45 yr (n= 44)**	
**Mites**	30.0	42.12	46.9	45.5	
**Molds**	27.5	30.3	16.7	25.0	
**Cockroach**	27.5	42.1	32.3	40.9	
**Weeds**	82.5	92.1	88.5	90.9	
**Grasses**	62.5	76.3	71.9	88.6*	
**Trees**	75.0	86.8	85.4	93.2	

* *p*<0.05.

Among outdoor allergens sensitization to *P*. *juliflora* and *E*. *globulus* [(*p*<0.04) and *p*<0.009), respectively], and among indoor allergens skin test reactivity to *D*. *pteronyssinus* (*p*<0.03) was significantly prevalent in allergic rhinitis patients with seasonal pattern (Table 3).

The mean total IgE serum was determined as 177.77 IU/ml. The mean total of IgE serum in men (174.31 IU/ml) was significantly greater than women (129.45 IU/ml) (*p*<0.01). Moreover, the mean total IgE serum among patients with positive SPT was significantly higher the one than in patients without positive SPT (177.77 vs. 4.56 IU/mL, *p* < 0.001).

## Discussion

Based on the results, 85.6% of patients with active rhinitis were responsive to at least one aeroallergen, which is found to be a higher percentage compared to other studies [1,15,16]. In the conducted study, 64.5% of patients with active rhinitis had family history of atopy (allergy), which is in agreement with some studies [3,15]. A family history of atopy is an established risk factor for allergy particularly allergic rhinitis [3,17].

The results of skin prick test in allergic rhinitis patients indicated the high incidence of allergy to plant pollens and the most common was found to be *S*. *kali* (*Russian thistle*) pollen with 72.9%. Allergy to *S*. *kali* pollen was reported as one of the most causes of allergic rhinitis in neighboring regions [6,18-21]. *Prosopis juliflora* (Mesquite) pollen (65.9%) was the most common sensitizing tree pollen in the present study. This is in accordance with previous studies in countries neighboring to our region [17,19,21]. Mesquite is abundant in Khuzestan and other region with hot and humid climate, where it is planted as shade and ornamental tree or for binding sand. Surprisingly, in spite of very limited presence of *Fraxinus americana* (Ash) tree in Khuzestan province, the Ash pollen was the second most common sensitizing tree pollen (52.5%) in our study. It may be due to confirmed cross-reactivity between pollens of ash and olive [22-24], a close taxonomical relationship tree, which is cultivated in orchards and sometimes in parks and gardens throughout the area. It is also possible that the high rate of sensitization to ash pollen is due to cross-reactivity among pollens of ash and grasses and weeds, as has been previously reported [22-24]. It could be supported by our findings that indicated a significant correlation between sensitization to pollens of ash and studied grasses or weeds (*p*<0.001) (data not shown).

Moreover, among other selected outdoor allergens, allergenic molds were found to be less prevalent, which in accordance to previous studies [9,17,21,25]. However, it seems that the exact prevalence of sensitization to molds is difficult depending on more variability of fungal antigens than other allergens, extracts used, and species tested [26,27].

Around 95% of patients with positive skin prick test were sensitive to at least one pollen type. This finding is also supported by studies conducted in other regions [3,5,15,25]. Recent studies have shown that high incidence of respiratory allergy is related to the global increase of CO2 and other greenhouse gasses, which leads to an increase in the production of pollens by plants, allergenicity of pollens, increase in production period and distribution of pollens, and changes in plant pattern in areas [28,29]. There is no doubt that environmental factors especially air pollution play an important role in the increasing allergies in the worldwide. In recent years, due to severe geographical and climate changing, the dust storm becomes a common phenomenon in our region [10-12]. To this date, there is no data available to report the effects of Middle East storm on public health particularly allergic diseases in this area. However, recently increasing evidence has been accumulated that exposure to particulate matters which contained various aeroallergens such as pollens and fungal spore, during dust storms could increase respiratory allergic diseases [11,12,30-32].

In recent years, due to climate change, the dust storm has become a common phenomenon in Khuzestan province [10-12]. To this date, there is no available data on the probable effects of Middle East storm on public health particularly allergic diseases in this area. Due to the lack of data, further studies are required to investigate the effect of dust storm on hospital visits, admission frequency, respiratory allergic diseases onset and mortality in local resident people.

In our research, prevalence of positive skin prick test to *Dermatophagoides farinae* and *Dermatophagoides pteronyssinus* was found to be 32.1% and 27.1% respectively, which was lower than the results of other studies [9,15]. Regarding to the optimum condition of 60% humidity and 25°C temperature, high incidence of mite allergy is expected in humid regions, such as Singapore [33], Malaysia [15] and Thailand [34]. Unexpectedly, this particular allergy was also found in hot and dry regions, like Sistan and Baluchestan province of Iran [9], Qatar [35] and Kuwait [17,21]. It may be associated with an increase in usage of air conditioners inside the houses which make good environment for mites to grow and increase exposure to indoor allergens [6,36,37].

In addition, prevalence of allergy to cockroach was determined to be 30.8%, which is higher than results of other studies [6,25,38,39], while the sensitization to cockroach allergens is one of the most common indoor allergens in some regions with similar weathers to our region [17,35,40].

The most of SPT positive patients (85%) were reactive to two or more allergens. This is in accordance with earlier reports [6,17,18,25]. It seems multi-allergen sensi-tization may be due to several factors, including cross-reactivity among allergens belonging to close reservoirs, which reflects the presence of common allergenic epitopes in different but botanically close plant species, long-term exposures to close phylogenic source of allergens, and interactions of genetic and environmental factors [6,17,18,25,37]. As the use of a panel of plant allergens belongs to closely related species; it may be an explanation of the high rate of multi-sensitization in our study.

In this study, no significant statistical relationship was found between the positive skin prick test and patient’s gender. Our data were similar with some studies [21,41], but in some others higher prevalence of allergy was seen in men compared to women [3,25,37,42]. This phenomenon cannot be explained since there is no evidence showing that men are more exposed to pollens than women. This is considerable since in some countries, such as Kuwait and Iran [17,43], men usually spend more time outdoors than women; therefore, they are more exposed to the outdoor allergens. However, our results show no significant difference between incidence of outdoor allergens and patient’s gender.

Traditionally, allergic rhinitis has been subdivided into seasonal and perennial types based on time and duration of symptom occurrence. In general, mites, cockroach and some molds are considered as perennial allergens, whereas weeds, grasses and trees are considered as seasonal allergens. In our studies, there was no significant correlation between selected allergens and pattern of allergenic rhinitis. The same results were obtained in some previous studies [44,45]. This may be due to several possible reasons: (1) it is difficult for patients to differentiate between seasonal or perennial allergies with the common cold and some active infections [44], (2) in tropical area with mild fall and winter, the exposure to some pollens is long standing because of increase in production period and distribution of pollens [28,29,44], (3) nasal inflammation is extended for weeks after pollen contact in patients with seasonal rhinitis [46], (4) the exposure to some perennial allergens is not comparable over the year and symptoms could be of short duration [44], and (5) in our study, the majority of patients (about 85%) are polysensitized to pollen and perennial allergens.

With exception of grasses, the analysis of date showed that there is no difference in the rates of positive skin prick test between younger and older allergic patients. However, some previous studies revealed that sensitization to some indoor allergens such as mites and cockroach is more common in younger patients [17,36]. It should be noted that in hot and dry climate area like Ahvaz city, the people spend most of their time inside buildings which have air conditioner, which may offer an explanation for high prevalence of indoor allergens in adults in present study [6,37].

In this study the mean total IgE serum in men was significantly higher than women (174.31 vs. 129.45) (*p*<0.01). Moreover, among skin prick test negative population, the average total IgE serum in men was greater than women. Elevated total IgE levels are usually associated to allergy, but it may be depend on various factors; such as parasitic infestations, smoking, pollution, local diet and different genetic background [25,47].

## Conclusions

The study revealed that prevalence of the skin prick reactivity to outdoor allergens, particularly weed pollens, is significant in southwest Iran and multiple sensitizations was surprisingly common. Moreover, exposure to components of dust storm such as pollens and fungal spores may affect human health directly through allergic induction of respiratory disorders. However, further studies are needed to identify components of dust particles and define association of this phenomenon with the prevalence or exacerbation of respiratory allergic diseases in local resident population.

## Competing interests

The authors declare that they have no competing interests.

## Authors’ contributions

MA, ASH and AA carried out data collection. MA and AA collated and analyzed statistics. MA drafted the manuscript. ASH finalized the manuscript. All authors read and approved the final manuscript.
